# Effect on health-related quality of life of a multimodal physiotherapy program in patients with chronic musculoskeletal disorders

**DOI:** 10.1186/1477-7525-11-19

**Published:** 2013-02-16

**Authors:** Antonio Ignacio Cuesta-Vargas, Manuel González-Sánchez, María Jesús Casuso-Holgado

**Affiliations:** 1 School of Clinical Science, Queensland University of Technology, Brisbane, Australia; 2 Physiotherapy Department, University of Malaga, Malaga, Spain

**Keywords:** Chronic musculoskeletal disorders, Multimodal physiotherapy, Aquatic exercise, HRQoL

## Abstract

**Background:**

Musculoskeletal disorders are major causes of morbidity in the world, and these conditions have a strong negative influence in terms of health-related quality of life. The purpose of this study was to evaluate the effect of an 8-week multimodal physical therapy program on general health state and health-related quality of life in patients with chronic musculoskeletal disorders.

**Methods:**

There were 244 participants in this prospective cohort analysis with 8-week follow-up. The primary outcome was general health state (physical and mental components), determined with the Short Form-12 Health Survey (SF-12). The secondary outcome was health related quality of life, determined with the EuroQoL-5D and VAS. The intervention was evaluated by comparing pre- and post-outcome measurements. T-tests were performed for paired data.

**Results:**

Differences were statistically significant for physical health state: +1.68 (p < 0.05) (baseline: 42.38); mental health state: +3.15 (p < 0.001) (baseline: 46.57); and health related quality of life: +0.18 (EuroQoL 5D) (baseline: 46.57) and +7.22 (EuroQoL_VAS) (p < 0.001) (baseline: 60.81). Intervention resulted in clinically relevant changes in terms of percentage improvement from baseline scores.

**Conclusions:**

Eight weeks of a Multimodal Physical Therapy Program seemed to moderately enhance the general health state and HRQoL of patients with chronic musculoskeletal diseases. This kind of therapeutic exercise can be recommended to patients with chronic low back pain, chronic neck pain and osteoarthritis, at least in the short term.

## Background

Musculoskeletal disorders (MSKD) are major causes of morbidity in the world and these conditions have a strong negative influence in terms of health-related quality of life [1]. In Europe, chronic musculoskeletal pain of moderate or severe intensity occurs in 19% of adults and these conditions can limit daily activities to a great degree [2]. It has been shown that people with musculoskeletal pain estimate their health-related quality of life very low compared to a pain free population, and that their perceived health can predict musculoskeletal pain outcome [3,4]. Musculoskeletal disorders have also been associated with more mental distress and depression [5]. In this way, one goal of the Bone and Joint Decade is to improve the health-related quality of life for people with, or at risk of, musculoskeletal disorders throughout the world by raising awareness of the suffering and cost to society associated with these conditions, by empowering patients to participate in decisions concerning their care, by promoting cost-effective prevention and treatment, and by advancing understanding of musculoskeletal conditions and improving prevention and treatment through research [1].

It is essential that the most effective methods to reduce pain in debilitating chronic diseases be used in their management [6]. There are a multitude of guidelines on how best to manage chronic musculoskeletal conditions [7-10], all stating exercise as a common treatment modality. Exercise is considered a part of effective physiotherapy treatment when it fulfils a number of criteria, e.g. efficacy, effectiveness, appropriate dose and mechanisms of action and avoidance of potential adverse effects [11]. A study investigating physiotherapy approaches to pain concluded that the restoration of movement and function make physiotherapy an essential part of the collaborative approach required for effective pain management [12].

The American College of Sports Medicine (ACSM) [13] recommends aerobic, strength, flexibility and functional components be included in exercise programme. For the chronic MSKD (CMSKD) relevant to this study, chronic low back pain (CLBP), chronic neck pain (CNP) and osteo-arthritis (OA), the ACSM guidelines recommend 2–3 treatment sessions per week. These could be incorporated in a multimodal exercise programme with psychological, educational and social aspects.

There is strong evidence to support the implementation of these programmes for persons with CMSKD [14-17]. Studies have also demonstrated that physical activity can improve quality of life in adults with CMSKD [18-20]; although most previously described studies refer to land-based treatments, another potential exercise medium is water. Buoyancy significantly decreases weight bearing and stress on weight bearing joints, bones, and muscles, thereby reducing pain. There is evidence suggesting that therapeutic aquatic exercise is beneficial to patients with CLBP [21,22] or OA [23]. However, compared with land-based interventions these effects are open to interpretation [24,25]. A multimodal programme has been successfully employed previously by Cuesta-Vargas *et al*., (2009; 2011; 2012) [26-29] on CLBP suffers, but no studio that uses a multimodal physiotherapy program (integrating therapeutic exercise and health education) in patients suffering CMSKD was found.

Just as there is a multitude of evidence suggesting that chronic pain has a negative impact not only on physical but also on psychological health and wellbeing [30], the outcome measures of an intervention ought to be multidimensional and include the subjective experience of the patient. This can be achieved using a health-related quality of life (HRQoL) measurement tool [31].

With these facts in mind, the purpose of the current study was to evaluate the effect of an 8-week multimodal physical therapy program (MMPTP) (integrating therapeutic exercise and health education) on general health state (physical and mental component) and health-related quality of life in patients with chronic musculoskeletal disorders.

## Methods

### Sample

This is a prospective cohort analysis of data aiming to determine whether there are differences in the outcomes of general health state and quality of life in persons with chronic musculoskeletal disorders of CLBP, CNP and OA after an 8-week MMPTP. Intervention was evaluated comparing pre- and post-outcome measurements. Data were collected between January 2008 and May 2012 in a community health centre in Torremolinos Spain.

All participants involved in the study adhered to the inclusion/exclusion criteria. Inclusion criteria: subjects between 18 and 65 years with a MSKD (low back pain, neck pain or osteoarthritis) diagnosed more than 12 weeks ago. The exclusion criteria were: refusal to participate, failure to provide written informed consent, irregular attendance, an infectious process, cancer with or without metastases, osteoporosis, fracture or changes in pharmacological treatment during the intervention period. Also, patients who showed cognitive impairment of any aetiology or intolerance to exercise or physical activity for any reason or had received radiation to the lower quadrant of the body within the previous 12 weeks from the start of the study were excluded. Specifically for CLBP and CNP, patients were excluded if they suffered pain in the spine as a result of specific spinal pathology, nerve root pain/radiculopathy or had a current exacerbation of pain. All participants provided written, informed consent and were provided with information on the procedures of the study. The study had ethics approval from the Consejería de Salud (Distrito Sanitario Costa Del Sol, Servicio Andaluz de Salud, Spain).

### Measurements

The primary outcome was general health state (physical and mental component), determined with the Short Form-12 Health Survey (SF-12) [32]. The secondary outcome was health related quality of life, determined with the Spanish version of EuroQoL-5D (EuroQoL-5D and EuroQol-VAS [33,34].

The SF-12 is a generic measure and does not target a specific age or disease group. It has been developed to provide a shorter yet valid alternative to the SF-36. The SF-12 is weighted and summed to provide easily interpretable scales for physical and mental health. Physical and Mental Health Composite Scores (PCS & MCS) are computed using the scores on twelve questions that range from 0 to 100, where a zero score indicates the lowest level of health measured by the scales and 100 indicates the highest level of health. Since all values are reported on a scale of 0–100, all changes can be understood in terms of absolute and relative percentages.

The standardized extended version of the EQ-5D was designed for the collection of health state values using a descriptive system and comprises the following 5 dimensions: mobility, self-care, usual activities, pain/discomfort and anxiety/depression. Each dimension has 3 levels: no problems, some problems, extreme problems. Total scores range from 1 to −1. The EQ VAS rating scale is a vertical 10 cm visual analogue scale, with the end points labelled best imaginable health state at the top and worst imaginable health state at the bottom having numeric values of 100 and 0, respectively. The reliability of the SF-12 is 0.70 to 0.89 [35,36], and that of the EuroQoL is 0.86 to 0.90 [34,37].

Subjects were assessed on the outcome measures at baseline and at 8-week follow-up by a group of 17 qualified, experienced physiotherapists to determine if short term benefits were gained.

### Interventions

A Multimodal Physical Therapy Program (MMPTP) (combining therapeutic exercise and health education) intervention was used and individualised to the participants. Each participant was interviewed and assessed individually by a physiotherapist (the therapists who assessed patients were different from the therapists who provided the treatment) at the start of their intervention using the ASETER 2.0 [38] computer program, which includes a number of relevant lifestyle questions. Subsequently an individualised programme of exercise was designed. The frequency of the programme was 2 times per week, involving 30 minutes of land-based and 30 minutes of aquatic-based tailored exercise. In addition, participants were also given chronic disease self-management information based on: postural care, physical activity, lifting weights, sedentary activities, sports, pain-free maximal physical activity level, behavioural advice, fear of movement, false beliefs and an active lifestyle.

Land-based exercises were composed of five minutes of mobility exercise, five minutes training of motor control and 20 minutes resistance and muscle strengthening exercises. Aquatic- based exercises consisted of aerobic exercise in the form of deep water running (DWR) at 55 to 85% age-predicted maximal heart-rate according to previous research [39]. DWR simulates running at the deep end of a pool aided by a flotation device that maintains the head above the water.

The programme continued for 8 weeks with participants being instructed and supervised in groups (10–12 subjects). Participants were encouraged to adopt an active role in the programme and the importance of compliance was emphasised.

### Data analysis and statistics

Data was grouped into patient categories and screened for any obvious errors, anomalies and duplications within the set. These were then clarified with the original research team.

Descriptive statistics, including measurements of central tendency and dispersion, were calculated for all outcome measurements. To establish whether there were any significant differences between the baseline data and that at the 8-week follow-up, t-tests for paired data were performed. F values were used to establish the over-all significance, as well as pair-wise comparisons to show where any significant values fell within the data set. We adopted p < 0.05 as significant and the data analysis was carried out using the Statistical Package for the Social Sciences (SPSS) (version 17.0 for Windows, Illinois, USA).

## Results

**Figure 1:**
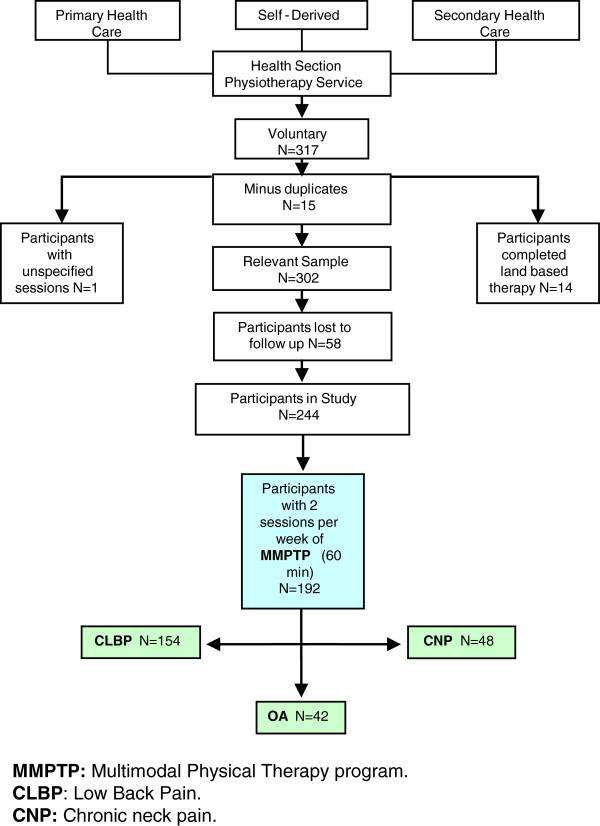
Figure to show progression of data analysis through study.

We identified 244 subjects for inclusion in the study (Figure 1). Fifty-six percent were female, with a mean age of 45.17 years old, (n = 154 suffered CLBP, n = 48 CNP, n = 42 OA). Descriptive statistics for the outcome measures at baseline are shown in Table 1. The degree of compliance of patients was 100% for those who began the study completing the treatment period, there being no casualties.

**Table 1:** Table to show descriptive statistics at baseline -central tendency and dispersion

**Outcome Measure**	**Mean (SD)**
AGE	**45.17** (±14.32)
BMI	**26.37** (±4.38)
SF12 – Phys	**42.38** (±10.48)
SF12 – Mental	**46.57** (±11.98)
EuroQol	**0.70** (±0.26)
EuroQol VAS	**60.81** (±22.65)

BMI: Body Mass Index.

SF-12 Phys: Physical Health State.

SF-12 Mental: Mental Health State.

EuroQoL: Mean Index from EuroQol 5D.

EuroQoL VAS: Visual Analogue Scale From EuroQoL 5DSD: standar desviation.

All data rounded to 2 decimal points.

The K-S test for normal distribution was completed on the baseline values representing parametric data. The mean differences and significance between baseline and 8 week follow-up scores are shown in Table 2.

**Table 2:** Differences and significance between baseline and 8 week follow-up using paired sample t-test

**Pathology**	**EQ-5D Basel**	**EQ-5D Post**	**EQ-5D Mean diff.**	**EQ-VAS Basel**	**EQ-VAS Post**	**EQ-VAS Mean diff.**	**SF-12 Phys basel**	**SF-12 Phys post**	**SF-12 Phys mean diff.**	**SF-12 Mental basel**	**SF-12 Mental post**	**SF-12 Mental mean diff.**
**CLBP**	0.76	0.95	0.19***	67.12	75.70	8.59***	43.16	44.9	1.74**	45.46	48.87	3.41**
(n = 154)	(±0.28)	(±0.21)	(0.04/-0.14)	(±23.00)	(±18.69)	(4.34/12.83)	(±10.42)	(±9.47)	(0.44/5.04)	(±11.90)	(±10.88)	(0.84/5.98)
**CNP**	0.70	0.80	0.1***	58.35	63.57	5.22***	42.80	43.45	0.65**	44.78	47.88	3.10***
(n = 48)	(±0.24)	(±0.18)	(0.05/0.14)	(±21.49)	(±19.67)	(4.38/14.06)	(±9.19)	(±9.50)	(0.31/2.98)	(±12.40)	(±9.93)	(2.66/7.54)
**OA**	0.55	0.75	0.20**	59.89	65.79	5.90***	38.41	41.86	3.45**	49.34	52.33	2.99***
(n = 42)	(±0.32)	(±0.28)	(0.02/0.27)	(±26.79)	(±22.02)	(6.71/21.29)	(±10.22)	(±10.42)	(0.63/6.26)	(±14.73)	(±10.42)	(3.22/10.76)
**CMSKD**	0.70	0.88	0.18***	60.81	69.03	7.22***	42.38	44.06	1.68*	46.57	49.72	3.15***
(n = 244)	(±0.26)	(±0.30)	(0.04/0.98)	(±22.65)	(±22.65)	(4.53/9.91)	(±10.48)	(±9.35)	(0.33/3.03)	(±11.98)	(±10.56)	(1.64/4.66)

**CLBP**: Chronic low back pain.

**CNP:** Chronic neck pain.

**OA:** Osteoarthritis.

**CMSKD**: Chronic musculoskeletal disease.

**EQ-5D:** EuroQoL 5 Dimensions.

**EQ-VAS:** EuroQoL visual analogue scale.

**SF-12 Phys:** Physical Health State.

**SF-12 Mental:** Mental Health State.

**Basel:** Baseline measurement.

**Post:** Post-intervention measurement.

Significance level:

* = p < 0.05.

** = p < 0.005.

*** = p <0.001.

Intervention resulted in an improvement in all outcome measures. Mean differences for general health state were 1.68 and 3.15 on the Physical and Mental components, respectively. Mean health related quality of life changed 0.18 points for EuroQoL and 7.22 for VAS.

Significance was found for the SF-12 Physical component (p < 0.005), SF-12 Mental component (p < 0.001), EuroQol (p < 0.001) and VAS (P < 0.001). Results of t-test for paired data showed that differences between baseline and 8 week follow-up scores were statistically significant in all outcome measurements. No significant differences in BMI were found comparing pre- and post-intervention measures.

## Discussion

The aim of the study was to determine whether a Multimodal Physical Therapy Programme of 8 weeks duration had any effect on improving general health state (physical or mental components) and health-related quality of life in patients with chronic musculoskeletal disorders. With clinical relevance considered as 10% improvement [40], such improvement was not observed in any case (%-%). However, t-tests for paired data showed that there were statistically significant differences between baseline and post-intervention scores in all outcome measurements.

All variables analysed in this study showed significant differences after eight weeks of intervention (Table 2). These differences were clinically relevant in the variable EQ-5D (0.18), and close to that limit in the variable EQ-VAS (7.22). For their part, the evolution in physical health state (PHS) and mental health state (MHS) could be interpreted as statistically significant maintenance of the states of physical and mental health to achieve improvements of 1.68 and 3.15, respectively (Table 2).

No studies have been done to analyse the evolution of HRQoL using MPTP as an intervention in several CMSKD simultaneously. However, some previous studies have been conducted in pathologies included in this study individually.Two studies have used MPTP treatment for subjects with CLBP [27,28]. In both studies the improvements observed in PHS and MHS were higher than those achieved in the present study. Thus, the participants improved PHS in 10.6 and 8.9 [27] 14.1 [28], 1.74 in the CLBP group and 1.68 in all participants of this study. These differences were also evident in MHS, with improvements of 22.1 [28], 3.41 in the CLBP group and 3.15 in the CMSKD group in this study. The explanation for these differences could be that the duration of treatment is longer, i.e. 15 [27], 12 [28] and 8 (present study) weeks.

Other studies have examined the effect of an intervention based on therapeutic exercise in the other two CMSKD and observed differences between the pre- and post-intervention measures comparable to the present study. Martel et al. [41] and Michalsen et al. [42] reported improvements in PHS and MHS of 6.1 and 4.2 [41] and 4.2 and 2.3 [42], respectively, after intervening CNP sufferers. These results, not being clinically relevant, could be interpreted as a maintenance of PHS and MHS in these patients, so they might be comparable to the results observed in the CMSKD (in general) and CNP (in particular) of the present study, who similarly achieved significant improvements of 0.65 and 1.68 in PHS and 3.10 and 3.15 in MHS, respectively.

On the other hand, two other studies that used exercise as a means of therapeutic intervention in patients with OA showed a significant maintenance of HRQoL. Frasen et al. [43] and Jigami et al. [44] observed how, in their intervention groups, patients achieved significant but not clinically relevant improvements of 4.0 and 0.9 in PHS and 0.9, in both studies, in MHS, respectively. These results are also comparable to those obtained in the OA group in this study, which achieved improvements of 3.45 and 2.99 (Table 2) in PHS and MHS, respectively. They are also comparable in the overall group CMSKD Sufferers, whose significant increases in PHS and MHS were 1.68 and 3.15, respectively.

Based on the results obtained in this study, and comparing them to other studies with similar characteristics, it is possible to affirm that a MPTP which integrates therapeutic exercise and health education achieved similar results if subjects are distributed by specific pathology groups or if they are integrated into a group of patients suffering CMSKD as presented in this study (CLBP, CNP and OA). This should be taken into account when planning and implementing a treatment program for these patients.

There is no control group is the most important limitation of this study. Beyond the lack of long-term follow-up, also indicated by Kamioka et al., (2010) [45] in relation to aquatic exercise research, should be taken into consideration for future research. The outcome measures used were on self-reported scales and thus very personal to each participant, depending upon that individual’s perception of quality of life and the values they place on aspects of their disease such as pain, physical/mental health etc. Individual perception of QoL will also change over time with different life experiences and aspirations. This very much depends upon the environment, culture and circumstances that one is exposed to. During the follow up sessions assessing the HRQoL, there was no indication as to any lifestyle changes encountered by the participants, or other variables such as changes in medication, exercise levels and changes within the grading of the participant’s chronic conditions that may have impacted on their scoring. These could all subsequently impact and affect the scoring of the outcome measures.

The SF12 as an outcome measure was chosen for its validity and practicality, yet it has been shown to yield less precise scores than the original SF36 outcome measure. However, the larger the sample size, as with this study, the smaller the effect of these differences, as the confidence intervals for group averages in health scores are largely determined by sample size.

Although the population sample was limited to persons from one region of Spain, the sample was fairly large and representative, and therefore the results are likely to be carried over to other regions and areas in Europe. It can be said that the results of the current study may be useful clinically, enabling physiotherapists to design an effective aquatic therapy programme and manage persons with chronic MSK disorders in a similar multi-modal intervention.

## Conclusion

Eight weeks of a Multimodal Physical Therapy Program seemed to moderately enhance the general health state and HRQoL of patients with chronic musculoskeletal diseases. This kind of therapeutic exercise can be recommended to patients with CLBP, CNP and OA, at least in the short term.

## Abbreviations

ACSM: American College of Sports Medicine; CLBP: Chronic low back pain; CNP: Chronic neck pain; DWR: Deep water running; EQ-5D: EuroQoL five dimensions; HRQoL: Health related quality of life; MCS: Mental component score; MMPTP: Multimodal physical therapy program; MSKD: Musculoskeletal disorders; OA: Osteoarthritis; PCS: Physical component score; QoL: Quality of life; SF-12: Short Form-12 Health Survey; SPSS: Statistical Package for the Social Sciences; VAS: Visual analogue scale.

## Competing interests

The authors declare they have no competing interests.

## Authors’ contributions

AIC-V participated in the design of the study and performed the statistical analysis and drafted the manuscript. AIC-V, MG-S collected the data and helped to draft the manuscript. MJC-H helped to draft the manuscript. All authors read and approved the final manuscript.
